# *In vitro* phytochemical analysis and antibacterial and antifungal efficacy assessment of ethanolic and aqueous extracts of *Rumex nervosus* leaves against selected bacteria and fungi

**DOI:** 10.14202/vetworld.2022.2725-2737

**Published:** 2022-11-29

**Authors:** Maged A. Al-Garadi, Mohammed M. Qaid, Abdulmohsen H. Alqhtani, Anthony Pokoo-Aikins, Saud I. Al-Mufarrej

**Affiliations:** 1 Department of Animal Production, College of Food and Agriculture Sciences, King Saud University, Riyadh, 11451, Saudi Arabia; 2 US National Poultry Research Center, Toxicology and Mycotoxin Research Unit, USDA, ARS, Athens, Georgia 30605, USA

**Keywords:** antimicrobial activity, *Aspergillus* spp, Gram-negative bacteria, Gram-positive bacteria, phytochemicals, *Rumex nervosus* leaves

## Abstract

**Background and Aim:**

Scientists are interested in identifying natural antibiotic substitutes that are effective against drug-resistant pathogenic microbes and spoilage fungi to counter pathogens and reduce the major public health problem of antibiotic residues in animal products. This study aimed to evaluate the antimicrobial activity of *Rumex nervosus* leaves (RNLs) as a medicinal herb against four bacterial and two fungal strains using absolute ethanol, 50% ethanol, and aqueous extracts.

**Materials and Methods:**

The antimicrobial activities of various RNL extracts against selected microbes were evaluated using the disk diffusion antibiotic susceptibility test, minimum inhibitory concentrations (MICs), minimum bactericidal concentrations (MBCs), minimum fungicidal concentrations, and the poisoned food technique.

**Results:**

The absolute ethanol RNL extract showed the best bacteriostatic/bactericidal activity against Salmonella *Typhimurium, Escherichia coli*, and *Staphylococcus aureus* (MIC/MBC: 0.20/0.40, 0.20/0.40, and 0.32/0.65 mg/mL, respectively). The diameter of the zone of inhibition was larger (p < 0.05) for the 100% ethanol RNL extract (8.17 mm) against *Salmonella Typhimurium*, the 50% ethanol-RNL extract (11.5 mm) against *E. coli*, and the aqueous RNL extract (14.0 mm) against *S. aureus* than for any other bacterial isolate. The aqueous RNL extract strongly (p < 0.0001) inhibited the mycelial growth of *Aspergillus fumigatus* (100%) and *Aspergillus niger* (81.4%) compared with the control.

**Conclusion:**

The results of this study suggest that RNL is a promising new natural antimicrobial agent for food preservation. To date, most research on the antimicrobial properties of natural herbs has been conducted *in vitro*, with few exceptions *in vivo* and intervention-based research.

## Introduction

Foodborne pathogens such as *Escherichia coli*, *Salmonella*, *Listeria monocytogenes*, and *Staphylococcus aureus* are among the hazards of major concern in the food sector worldwide [1]. Due to their potential pathogenicity, they have a significant impact on public health, on the cost of treating these infections, and on the economic consequences for exporters of food or meat. *Listeria monocytogenes* has a high mortality rate (about 20%) in humans and is considered as one of the most serious foodborne diseases [2]. Between 1999 and 2019, *Salmonella* was the most common foodborne pathogen in African food exports to the European Union [3]. *Staphylococcus aureus*, *Bacillus cereus*, *Yersinia enterocolitica*, parasites, and other pathogens have been reported to cause illness [1]. Between 1998 and 2008, poultry products accounted for the most foodborne illness and human deaths in the United States out of 17 commodities [4]. Fungal contamination in food, feed, and other agricultural commodities leads to massive spoilage or decay and a range of food safety problems [5]. In addition, mycotoxins are secondary fungal metabolites that are potentially toxic and pose a high risk of ergot poisoning, aflatoxicosis, leukocyte deficiency, and other human diseases [6, 7]. Although therapeutic antibiotics are available against infectious diseases, many researchers throughout human history have been interested in treating a wide range of infectious diseases and pain with herbal remedies [8–10]. However, antibiotic resistance is a major public health problem caused by antibiotic residues in animal products. The widespread, excessive, and indiscriminate use of commercially available antimicrobial drugs to cure infectious diseases has led to the emergence of antibiotic-resistant bacteria [11, 12]. Therefore, scientists are searching for new sources, such as natural antimicrobial agents (phytocompounds) that are active against drug-resistant pathogenic microbes and spoilage fungi to counteract pathogens and reduce these problems [13].

Various medicines or active ingredients are extracted from herbal and traditional medicines to treat diseases, improve health, and support body functions [14]. Essential oils (EOs) from garlic, onion, and cinnamon show effective anti-biofilm activity against *L. monocytogenes* and are promising natural antimicrobial alternatives for food processing plants [15]. As a result, medicinal plants have become increasingly important in maintaining global health [13, 16]. Researchers are interested in using natural antioxidants found in plants and other biological materials because they are considered safe and effective in overcoming pathogen and nutrient resistance, as well as therapeutically helpful [17]. Due to their extensive biological and therapeutic properties (bioactive compounds), herbal medicines are an important source of health care in both developed and developing countries [13]. According to recent statistics from the World Health Organization, herbal medicines and dietary supplements are used by more than 80% of people worldwide as part of their primary health care [18]. The antimicrobial activity of medicinal plants needs to be tested to identify potential new compounds for therapeutic use [19]. *Rumex nervosus* is a medicinal shrub used throughout Asia and North Africa to cure a variety of ailments, such as inflammatory diseases [20, 21]. *Rumex nervosus*, a plant used in folk medicine, was selected by Yemenis [21, 22] and Saudis [23] to test its antimicrobial activity. It contains active compounds such as gallic acid, which can be used to treat chronic and infectious diseases and inflammatory conditions [24]. Although *R. nervosus* possesses antibacterial, antioxidant, and anti-inflammatory properties [25], its potential as an antifungal agent remains to be tested. Although the antimicrobial activity of *R. nervosus* has been investigated, a comparison of the activity of ethanol extracts of *R. nervosus* leaves (RNL) with different degrees of polarity (100%, 50%, and 0% ethanol) at high concentrations has not yet been studied in detail.

Therefore, this study aimed to evaluate and compare the different RNL extracts for their antimicrobial activity against four bacteria (*S. aureus*, *L. monocytogenes*, *E. coli*, and *Salmonella enterica* subspecies Typhimurium) and two fungal isolates (*Aspergillus fumigatus* and *Aspergillus niger*).

## Materials and Methods

### Ethical approval

This study does not require animal ethics approval because this study was conducted *in vitro* without using humans, animals, or tissues derived from them.

### Study period and location

The study was conducted from February to November 2020. This study was conducted in Poultry Health Lab., at the Animal Production Department, King Saud University (KSU), located at Riyadh, Saudi Arabia, (24.92°N latitude and longitude of 46.72°E, 600 meters above sea level). In this region, the average annual rainfall is 110.6 mm, and the mean temperature of 26.4°C with a humidity of 65%.

### Preparation of RNL powder

*Rumex nervosus* leaves were collected from the valleys and mountains around Bait Al-Aqra village in Ibb region of Yemen. RN exsiccate registration No. 23033, registered by botanist Dr. Jacob Thomas at King Saud University’s Department of Botany, Faculty of Science. The harvested RNL was air-dried under sunlight every 5 h for 15 days. Then, the dried leaves were ground into a fine powder (particle size; 0.25–0.30 mm) using a blender (Nima Electric Super Speed Dry Spices Grinder) in our laboratory.

### Preparation of RNL extracts

The ground RNL powder extracts for the antimicrobial tests were prepared according to the method described by Muthuswamy *et al*. [26]. *Rumex nervosus* leaves extracts were prepared using three solvents: Absolute ethanol (R100EOH), 50% ethanol (R50EOH), and pure distilled water (DW) alone [Rumex water extract (RWA)]. Then, 150 g of RNL powder was weighed and distributed to each vials (50 g/vial). Each vial was thoroughly mixed in a tightly stoppered flask and dissolved separately at a ratio of 1:10 (w/v) in 500 mL sterile DW for water extraction, 500 mL ethanol/water (2:2, v/v) for ethanol/water extraction, and 500 mL absolute ethanol for ethanol extraction. The flasks were then boiled under reflux at 37°C in a water bath for 30 min, 45 min, and 5 h, respectively. The supernatants were then collected and filtered through 0.22 mm Millipore membrane filters (Whatman filter paper # No. 1) into new sterile flasks. The filtrate was then dried completely or evaporated at 45°C in an oven or with a rotary evaporator (Eyela, Irvine, California, USA). Before *in vitro* antimicrobial assays, the dried herbal extracts were weighed and dissolved in dimethyl sulfoxide (DMSO) to obtain stock concentrations of 500 mg/mL of each crude plant extract or stored in sealed bottles for later use.

### Percentage yield of RNL extraction (% yield)

The yield (%, w/w) of each dry extract was calculated according to the following formula: Yield (%) = (W_1_ *100)/W_2_, where W_1_ is the weight of extract after drying with solvent (lyophilization) and W_2_ is the weight of RNL powder before extraction [27].

### Determination of the total phenolics of RNL

The Folin–Ciocalteu colorimetric method was used to determine the concentration of total phenols in an ethanol extract of RNL powder [28]. For the extract, 25 mL of ethanol was added to 1 g of RNL powder and allowed to stand overnight at room temperature (24°C). A 200 mL of the ethanol solution was pipetted into a flask. Then, 4 mL of DW and 1 mL of Folin–Ciocalteu reagent (1 N) were added and shaken for 10 min. After 3 min, 1.0 mL of 20% sodium carbonate was added to the mixture. After 1 h of incubation at 24°C and shaking, absorbance was measured using a spectrophotometer at 760 nm. Measurements were performed in duplicate, the calibration curve was prepared using gallic acid, and the results were expressed in gallic acid equivalent (GAE)/mg.

### Phytochemical analysis (gas chromatography–mass spectrometry [GC–MS] and high-performance liquid chromatography [HPLC]) and proximate analysis of RNL

High-performance liquid chromatography was used to identify, separate, and dosing of chemical compounds in the RNL extract mixture. Gas chromatography–mass spectrometry assay was used to determine the chemical composition as described by Adaszyńska-Skwirzyńska and Szczerbińska [29]. To determine the macronutrients and nutritional value of RNL, proximate analysis was performed as described by Melesse *et al*. [30].

### Antibacterial activity of RNL extracts

#### Microorganisms and preparation of bacterial inoculum

The reference strain of two Gram-positive bacteria: *S. aureus* ATCC 29737 and *L. monocytogenes* ATCC 13932 and two Gram-negative bacteria: *E. coli* ATCC 25922 (*E. coli*) and *S. enterica* subspecies Typhimurium ATCC 14026 were obtained from standard stocks in our laboratory, College of Food and Agriculture, King Saud University.

Bacterial inoculum was prepared according to the standard protocol of the Clinical and Laboratory Standards Institute (CLSI) [31]. The bacterial stock cultures were activated by suspension in nutrient broth for 24 h before subculturing on sterile nutrient agar plates. To ensure that the bacteria were fully activated, sub cultivation was repeated 3 times. The inoculum suspension for each assay was prepared by taking 4 or 5 pure colonies from a fresh culture. The colony suspensions were emulsified in sterile saline and successively diluted until turbidity was comparable to a 0.5 McFarland turbidity standard. This resulted in inoculum density of nearly 10^8^ colony-forming units (CFU)/mL.

#### Screening for antimicrobial activity (disk diffusion antibiotic susceptibility test)

The RNL extracts (absolute ethanol, 50% ethanol, and water extract) were screened for antibacterial activity against various bacteria (*S. aureus*, *L. monocytogenes*, *E. coli*, and *Salmonella* Typhimurium) using the agar disk diffusion method (ADDM) based on the standard guidelines of the National Committee for Clinical Laboratory Standards [32] with minimal modifications. After adjusting the turbidity of the inoculum suspension using McFarland turbidity standards, the adjusted microbial suspensions were carefully blotted onto sterile Mueller-Hinton agar plates using a sterile cotton swab. The inoculum was dried in a safety cabinet (Class II biological safety cabinet from NuAire, Plymouth, MN, USA) for 30 min. Circular antibiotic test disks of 6 mm diameter were prepared using Whatman filter paper (No. 3). Following Alhajj *et al*. [33], the sterile dried disks were aseptically applied to the inoculated agar plates after being soaked in 40 mL of the respective RNL extract. The plates were left at 4°C for 1 h before incubation at 37°C for 24 h. The diameter of the inhibition zones (DIZ) was then measured (in mm). DIZs of <12, 12–16, and >16 mm were classified as having weak, moderate, and strong antibacterial activity, respectively [34]. Microbes were tested for susceptibility to the standard antibiotic piperacillin (100 mg) as a positive control (PC). Paper disks soaked with the appropriate extractant (DMSO) were used as negative control (NC). The tests were performed in triplicate.

#### Determination of minimum inhibitory concentration (MIC) and minimum bactericidal concentrations (MBC) of the tested bacteria

The CLSI method and the microdilution technique (a microplate method) were used to determine the MIC of the RNL extracts [35]. The MIC was determined by selecting the lowest concentration of each RNL extract that did not cause visible bacterial growth [36]. The bacteria in the adjusted inoculum suspensions were further diluted (1:100) to 10^6^ CFU/mL. The assay was performed as described by Bekkar *et al*. [37]. Wells with absorbance values comparable to the PC indicated turbidity or bacterial growth, while wells with the lowest extract concentrations and absorbance comparable to the NC indicated complete growth inhibition and were considered MIC values.

MBC is defined as the lowest antimicrobial concentration required to completely inhibit bacterial growth (without bacterial turbidity). To calculate MBC, 10 μL of inoculum from wells without bacterial growth was inoculated into sterile nutrient agar plates. Plates inoculated with bacteria served as PC, while plates containing only nutrient agar served as NC. Wells that did not show growth were used to determine MBC. Bacterial growth was measured after incubation at 37°C for 48 h. The experiment was performed in triplicate.

### Antifungal activity

To test the activity of RNL extracts against the pure fungal strains *A. niger* ATCC 6275 and *A. fumigatus* ATCC 28282, the ADDM, microtiter plate method, and poisoned food technique were used. The MIC and minimum fungicidal concentrations (MFCs) were also determined. *Aspergillus fumigatus* and *A. niger* were obtained from the Food Microbiology Laboratory of the Department of Food and Nutritional Sciences, College of Food and Agriculture Sciences, King Saud University.

#### Preparation of fungal spores

The fungal suspension was prepared according to the methodology of the European Committee on Antimicrobial Susceptibility Testing-Antifungal Susceptibility Testing Subcommittee against *Aspergillus* spp. (EUCAST-AST-*ASPERGILLUS*) [38]. Briefly, fresh, mature (2–5 days old) cultures were grown on sterile potato dextrose agar at 35°C to prepare inoculum suspensions. Colonies were covered with 1 mL of sterile water, to which 0.1% Tween 20 was added. The conidia were collected rationally with a sterile cotton swab and transferred to a sterile tube. The inoculum was homogenized with a rotating vortex blender at 376 × g for 15 s. Then, the conidia were diluted with sterile water, counted in a hemocytometer and examined for hyphae and clumps. If a significant number of hyphae or clumps were present (>5% of fungal structures), the inoculum was filtered through a sterile nylon mesh filter with a pore size of 11 mm. Then, the suspension was adjusted to 2–5 × 10^6^ CFU/mL with sterile DW by counting the conidia in a hemocytometer. This suspension was then further diluted 1:10 with sterile DW to obtain a final working inoculum of 2–5 × 10^5^ CFU/mL. All adjusted suspensions were quantified by plating on SDA plates.

Miconazole nitrate (20 mg/g) was dissolved in DMSO at a concentration of 20 mg/mL and stored at −20°C. The stored solutions were then diluted to the concentrations used in this study. The final concentration of DMSO in the reaction mixture was limited to <1% (v/v) [39].

#### Antifungal susceptibility testing using the disk diffusion method

This technique was used to determine the suppressive activities of RNL extracts against *A. niger* and *A. fumigatus*, according to Naeini *et al*. [40]. Briefly, 10 mL of an adjusted suspension of *A. niger* or *A. fumigatus* was evenly distributed on potato dextrose agar plates. The inoculated plates were dried at 24°C in a safety cabinet. Then, blank paper disks with a thickness of 5 mm were impregnated with 40 μL of selected herbal extracts on the surface of the previously inoculated plates. The plates were sealed with parafilm and incubated at 35°C for 48–72 h. Each plate was examined after 48–72 h of incubation. The DIZ, as well as the diameter of the disk were measured. Miconazole nitrate and DMSO-soaked paper disks were used as positive and NC disks, respectively. The experiment was performed in triplicate.

#### Determination of MICs and MFCs of the tested fungi

Minimum inhibitory concentrations of RNL extracts on *A. niger* and *A. fumigatus* were determined using a microplate method as described by Lass-Flörl *et al*. [38]. The adjusted inoculum suspensions (containing conidia or sporangia spores) were mixed with a vortexer (2–5 × 10^5^ CFU/mL). *Rumex nervosus* leaves extracts were dissolved in DMSO (10% of the final volume) and diluted to a concentration of 500 mg/mL. Sterile 96-well apartment microtiter plates (Nunclon, Denmark) were used for this assay. Each well contained 100 mL of sterile Roswell Park Memorial Institute Medium 1640 with 2% glucose as a test medium. Then, serial 2-fold dilutions (the concentration range obtained was between 250 and 0.24 mg/mL) of each stock solutions of the extracts (500 mg/mL) were performed. Then, except for the NC, 100 mL of the adjusted inocula were added to each well. The diluted inoculum suspensions without extracts served as active controls, and the medium without extracts was used as NC. The plates were incubated for 48 h at 35°C in the incubator. The MIC was defined as the lowest concentration of extracts in the wells that showed no visible growth.

From the wells that showed no growth, 10 μL were taken and inoculated onto potato dextrose agar plates for the determination of MFC. The plates inoculated with fungi served as PCs, and the plates with potato dextrose agar alone served as NC. Plates were incubated at 35°C for 7 days, and fungal growth was assessed. The MFC was defined as the highest dilution (lowest concentration of extracts) at which no growth occurred on the plates. The test was carried out in three replicates.

#### Evaluation of the antifungal activity of RNL extracts by poisoned food technique

According to Shrestha and Tiwari [41], the antifungal activity of the extracts was evaluated by the poisoned food technique. A 0.5 mL of each extract (250 mg/mL concentration) was poured into the Petri plate, followed by the addition of 9.5 mL of sterile melted potato dextrose agar medium to a final volume of 10 mL and then gently swirled to achieve thorough mixing of the contents. After solidification, a 5 mm diameter *A. niger* or *A. fumigatus* inoculum disk was aseptically inoculated upside down into the center of the plates and incubated at 30°C for 7 days. The plates with potato dextrose agar without extracts served as NC, and the plates with miconazole nitrate served as PC. Subsequently, the average diameters of fungal growth were measured on the 7^th^ day of incubation and then used to calculate the inhibition of mycelial growth [42]. The percentage (%) of inhibition of mycelial growth was determined as [(g_c_–g_t_)/g_c_] × 100, where g_c_ is the average increase in mycelial colony growth in the NC and g_t_ is the average increase in mycelial colony growth on the plates treated with the selected extracts.

### Statistical analysis

Statistical Analysis System software 9.4 (SAS Institute Inc., NC, USA) Statistical Analysis System [43] was used to analyze the *in vitro* data. Antibacterial and antifungal efficacy was expressed as mean ± standard deviation, and one-way variance analysis of variance(ANOVA) was used to distinguish between groups. Means of data were analyzed for statistical differences using Duncan’s new multiple range test. p < 0.05 was considered statistically significant.

## Results

### Extracts yield

The extraction solvent had a significant effect on the extract yield. For each extraction, 150 g of RNL powder was extracted and concentrated in an oven at 45°C. The final weight of the extracts was 32.00, 33.00, and 34.20 g for R100EOH, R50EOH, and RWA extracts, respectively, corresponding to a percent extract yields of 21.23, 22.00, and 22.80 (w/w).

### Total phenolic content

The total phenols in the leaves of *R. nervosus* were 56.31 μg/mg dry weight. The total phenolic content in the ethanolic extract was 171.15 mg GAE/g for RNL.

### Nutrient analysis and phytochemical composition of RNL powder

As shown in Table-1, RNL contained 94.3% dry matter or 5.67% moisture. *Rumex nervosus* leaves contained 82.0% organic matter and 18.01% inorganic matter (crude ash). In addition, RNL contained 8.24% crude fiber, 13.6% crude protein, and 1.54% ether extract. The acid detergent fiber content was 15.5% and the neutral detergent fiber content was 20.2%. The nutritional value was 3273.31 calories. The results of proximate analysis of the dried leaves of *R. nervosus* indicate that they have the potential to be used as a dietary supplement.

**Table-1:** Proximate composition of dried leaves of *Rumex nervosus*^1^

Parameter	As-fed basis (%)
Moisture	5.67
Dry matter	94.33
Inorganic matter (crude ash)	18.01
Organic matter	81.99
Crude protein	13.63
Ether extract	1.54
Nitrogen-free extract (NFE)	58.58
Total crude fiber	8.24
Total carbohydrate	52.91
Total fiber fractions	
A. Acid detergent fiber	15.48
B. Neutral detergent fiber	20.21
Nutritive value (gross energy ((Calories)	3273.31

^1^The values are the results of a triplicate chemical analysis; NFE or digestible carbohydrates = 100– (protein+lipid+ash+fiber); Total carbohydrate = 100 − (% ash+% moisture+% crude fiber+% crude protein); Organic matter = 100 - Inorganic matter

**Figure-1:**
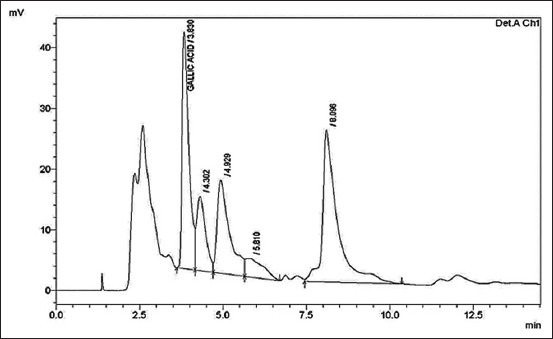
High-performance liquid chromatography chromatogram of the standard mixture of flavonoids and phenols of *Rumex nervosus* at 280 nm.

**Figure-2:**
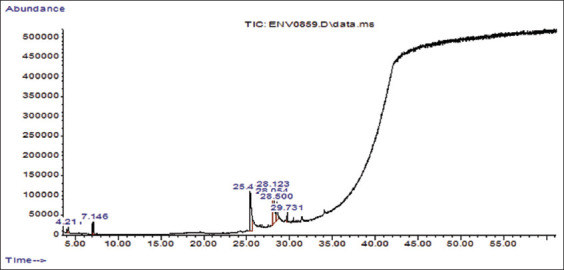
Gas chromatography–mass spectrometry tracing of *Rumex nervosus* leaves extract.

According to the HPLC results, gallic acid was the constituent with the highest concentration (700 mg/g) in RNL extracts (Table-2 and Figure-1). The constituents of RNL identified in Table-3 and Figure-2 were analyzed using GC–MS. The 11 volatile compounds were observed to be the most abundant compounds. As shown in Table-3, octadecanoic acid (98%), hexadecanoic acid (98%), pentadecanoic acid (97%), tridecanoic acid (86%), and nonadecanoic acid (53%) are present in RNL. Polyunsaturated fatty acids in RNL include 10, 13-octadecadienoic acid (99%), 9,12-octadecadienoic acid (99%), and 9,17-octadecadienal (90%). In addition, oxime-methoxy-phenyl (74%), cyclotrisiloxane, hexamethyl- (72%), 5-methyl-2-phenylindolizine (59%), and 1,2-bis(trimethylsilyl)benzene (50%) were detected at GC–MS.

**Table-2:** Individual flavonoids and phenols (g/g) in RNL powder extract were analyzed using HPLC

Compound	Retention time (RT) (min)	Area	Height	Concentration (µg/g)
Gallic acid	3.830	554308	38945	700
Catechin	-	-	-	-
Chlorogenic acid	-	-	-	-
Caffeine	-	-	-	-

**Table-3:** The main compounds (%) detected by gas chromatography-mass spectrometry (GC-MS) in the RNL powder extract

Retention Time (RT) (min)	Bioactive chemical constituents	Lipid number (Carbon & Bound)	Quality (%)	Molecular weight (amu)	Molecular Formula
28.50	Octadecanoic acid	C18:0	98	284.48	C_18_H_36_O_2_
28.12	10,13-Octadecadienoic acid	C18:2n-5	99	280.229	C_18_H_32_O_2_
28.12	7-Pentadecyne	C15:0	96	208.219	C_15_H_28_
28.05	9,12-Octadecadienoic acid (Z, Z)-	C18:2n−6	99	280.45	C_18_H_32_O_2_
28.05	9,17-Octadecadienal, (Z)-	C18:2n−1	90	264.245	C_18_H_32_O
25.41	Hexadecanoic acid	C16:0	98	256.4241	C_16_H_32_O_2_
25.41	Pentadecanoic acid	C15:0	97	242.40	C_15_H_30_O_2_
25.41	Tridecanoic acid	C13:0	86	214.34	C_13_H_26_O_2_
25.41	Nonadecanoic acid	C19:0	53	298.50	C_19_H_38_O_2_
7.146	5-Methyl-2-phenylindolizine	C15:0	59	207.105	C_15_H_13_N
7.146	1,2-Bis (trimethylsilyl) benzene	C12:0	50	222.126	C_12_H_22_Si_2_
7.026	Cyclotrisiloxane, hexamethyl-	C6:0	72	222.056	C_6_H_18_O_3_Si_3_
4.211	Methoxy phenyl oxime	C8:0	74	151.063	C_8_H_9_NO_2_

### Antibacterial activity

#### Screening for antimicrobial activity

The activities (growth inhibition zones) of the absolute ethanolic, 50% ethanolic, and aqueous extracts of RNL plant on the bacterial isolates (*S. aureus*, *L. monocytogenes*, *E. coli*, and *Salmonella* Typhimurium) by ADDM are shown in Table-4. Compared to standard antibiotics (piperacillin), the extracts had the lowest antibacterial activity. Piperacillin has been reported to have similar antimicrobial activity against these organisms when used as a standard. As expected, the antibiotic used produced the highest DIZ of 17.67 ± 1.7, 16.17 ± 1.3, 19.33 ± 0.9, and 17.83 ± 0.2 for *Salmonella* Typhimurium, *E. coli*, *S. aureus*, and *L. monocytogenes*, respectively. In this test, zones of inhibition >6 mm in diameter were considered positive. Six extracts from two different plant species were found to have different antimicrobial activities against the tested bacteria.

**Table-4:** Zone of inhibition (mm) of *Rumex nervosus* leaves extracts against selected bacteria

Microorganism	R100EOH	R50EOH	RWA	Piperacillin	SEM	*P*-value
*Salmonella* Typhimurium ATCC 14026	8.17±0.8^bA^	10.67±1.3^bAB^	8.50±1.3^bB^	17.67±1.7^a^	0.829	0.0001
*Escherichia coli* ATCC 25922	7.17±0.3^cAB^	11.50±0.5^bcA^	8.50±0.5^cB^	16.17±1.3^a^	0.514	<0.0001
*Staphylococcus aureus* ATCC 29737	6.67±0.8^dB^	9.00±1.0^cBC^	14.00±1.0^bA^	19.33±0.9^a^	0.571	<0.0001
*Listeria monocytogenes* ATCC 13932	6.77±0.3^bB^	7.23±1.5^bC^	6.83±0.6^bB^	17.83±0.2^a^	0.487	<0.0001
Standard error mean (SEM)	0.331	0.658	0.527	0.833		
*P*-value	0.044	0.008	<0.0001	0.142		

R100EOH: 100% Ethanol; R50EOH: 50% ethanol; and RWA: Water alone *Rumex nervosus* leaves extracts; Values are mean±standard deviation of a triplicate experiment; ^a–d^Rows with different letters indicate statistically significant differences (p < 0.05). ^A–C^Columns with different letters indicate statistically significant differences (p < 0.05).

Depending on the sensitivity of the bacteria tested, all bacterial strains were sensitive to RNL extracts to varying degrees. However, the RNL extracts had no significant effect on *L. monocytogenes* isolates. R100EOH showed the least antibacterial activity among the extracts, with a DIZ of 6.7–8.2 mm. However, RWA was superior to R100EOH and R50EOH against *S. aureus*. Antibacterial inhibitory activity against *S. aureus* increased with increasing polarity (inhibition zone 6.7, 9.0, and 14.0 mm for R100EOH, R50EOH, and RWA, respectively). The diameter of the zone of inhibition was larger (p < 0.05) for R100EOH against *Salmonella* Typhimurium (8.17 mm), R50EOH against *E. coli* (11.50 mm), and RWA against *S. aureus* (14.00 mm) compared to other bacteria tested.

#### Microplate dilution test

The MIC and MBC values of the tested medicinal plant extracts against the studied microbial strains are shown in Table-5. The RNL extracts have bacteriostatic and bactericidal effects on the tested microbes at these MIC and MBC concentrations. R100EOH showed antibacterial activity in this study with MIC and MBC values of 0.20–10.41 mg/mL and 0.40–20.81 mg/mL, respectively. The lowest MIC and MBC values were observed for *E. coli* and *Salmonella* Typhimurium, followed by *S. aureus*, while *L. monocytogenes* showed the highest MIC and MBC values. R50EOH also showed antibacterial activity with MIC and MBC values of 0.82–15.63 mg/mL and 1.63–31.26 mg/mL, respectively. The MIC and MBC values for R50EOH were lower for *Salmonella* Typhimurium than for *S. aureus* and lower for *E. coli* than for *L. monocytogenes*. In addition, RWA showed antibacterial activity with MIC and MBC values of 6.51–7.81 mg/mL and 13.02–15.62 mg/mL, respectively. The MIC and MBC values for RWA were similar for all bacteria tested with 6.51 mg/mL and 13.02, respectively, except for *L. monocytogenes*, which had 7.81 and 15.62 mg/mL, respectively.

**Table-5:** Minimum inhibitory concentrations (MIC) and minimum bactericidal concentrations (MBC) values for *Rumex nervosus* leaves extracts on *Salmonella* Typhimurium ATCC 14026, *Escherichia coli* (ATCC 25922), *Staphylococcus aureus* (ATCC 29737), *Listeria monocytogenes* (ATCC 13932)

Bacterial type/Extracts	R100EOH	R50EOH	RWA	SEM	Probability
Minimum inhibitory concentrations (MIC)					
*Salmonella* Typhimurium	0.20±0.1^b^	0.82±0.3^b^	6.51±2.3^a^	0.757	0.002
*Escherichia coli*	0.20±0.1^b^	2.60±1.1^b^	6.51±2.3^a^	0.835	0.005
*Listeria monocytogenes*	10.41±4.5^b^	15.63±0.0^a^	7.81±0.0^b^	1.501	0.027
*Staphylococcus aureus*	0.32±0.2^b^	2.64±1.1^b^	6.51±2.3^a^	0.837	0.006
Minimum bactericidal concentrations (MBC)					
*Salmonella* Typhimurium	0.40±0.1^b^	1.63±0.6^b^	13.02±4.5^a^	1.513	0.002
*Escherichia coli*	0.40±0.1^b^	5.21±2.3^b^	13.02±4.5^a^	1.669	0.005
*Listeria monocytogenes*	20.81±9.0^b^	31.26±0.0^a^	15.62±0.0^b^	3.002	0.027
*Staphylococcus aureus*	0.65±0.3^b^	5.27±2.20^b^	13.01±4.5^a^	1.637	0.006

R100EOH: 100 percent ethanol, R50EOH: 50 percent ethanol, or RWA: water alone are used to extract *Rumex nervosus* leaves. SEM: Standard error mean; Means of triplicates±standard deviation. The lower the MIC or MBC value, the better. ^a–b^Rows with different letters indicate statistically significant differences (p < 0.05). The concentrations of each extract were in the range of 500–0.12 mg, 500 mg (1), 125 mg (2), 62.5 mg (3), 31.3 mg (4), 15.6 mg (5), 7.81 mg (6), 3.91 mg (7), 1.95 mg (8), 0.98 mg (9), 0.49 mg (10), 0.24 mg (11) and 0.12 mg (12).

Based on the MIC and MBC values of the selected bacteria, the lowest MIC and MBC values were observed when R100EOH was tested with *E. coli* and *Salmonella* Typhimurium. Thus, the result revealed that the extracts obtained from RNL, particularly the absolute ethanol, are the most active extracts. The extracts had the lowest MIC and MBC values against the tested bacteria, except for *L. monocytogenes*, which were very resistant to RNL extracts. *Rumex nervosus* leaves extracts had an antimicrobial activity with bactericidal properties observed at different concentration ranges against all clinical bacteria tested in this study. The results suggest that the phenols, flavonoids, and tannins present in RNL extracts may have increased toxicity against pathogenic microorganisms.

### Antifungal activity

The antifungal activity of RNL extracts against the tested fungi (*A. fumigatus* and *A. niger*) using the ADDM and microtiter plate methods is shown in Table-6. The antifungal activity of three RNL extracts tested for *A. fumigatus* and *A. niger* showed no practical activity in the sensitivity test. R100EOH had no effect on the fungal growth of *A. fumigatus* and *A. niger* when tested with ADDM, but it was the most fungi static and fungicidal when tested with MIC and MFC.

**Table-6:** *In vitro* antifungal activity of *Rumex nervosus* leaves extracts against *Aspergillus fumigatus* ATCC 28282 and *Aspergillus niger* ATCC 6275

Fungal type/Extracts	R100EOH	R50EOH	RWA	Miconazole	SEM	Probability
Inhibition zone (mm)			
*Aspergillus fumigatus*	6.0±0.0^b^ (R)	6.0±0.0^b^ (R)	6.0±0.0^b^ (R)	15.0±0.82^a^	0.289	<0.0001
*Aspergillus niger*	6.0±0.0^b^ (R)	6.0±0.0^b^ (R)	6.0±0.0^b^ (R)	20.0±1.87^a^	0.661	<0.0001
Minimum inhibitory concentration (MIC) (mg/mL)			
*Aspergillus fumigatus*	26.1±7.4^b^	62.5±0.0^a^	62.5±0.0^a^	-	3.292	0.0009
*Aspergillus niger*	31.3±0.0^b^	62.5±0.0^a^	62.5±0.0^a^	-	5.763	<0.0001
Minimum fungicidal concentration (MFC) (mg/mL)			
*Aspergillus fumigatus*	52.1±14.7^b^	125±0.0^a^	125.2±0.0^a^	-	6.585	0.001
*Aspergillus niger*	62.5±0.0^b^	125±0.0^a^	125.0±0.0^a^	-	0	<0.0001

R100EOH: 100 percent ethanol, R50EOH: 50 percent ethanol or RWA: water alone are used to extract *Rumex nervosus* leaves. Means of triplicates±standard deviation; The lower the MIC or MFC, the better. ^a–e^Column with different letters indicate statistically significant differences (p < 0.05). R, resistance only disk zone “6 mm” without inhibition. (-) no tested. SEM: Standard error mean.

The inhibitory effects of the three RNL extracts on the mycelial growth of selected fungi using the food poisoning method are presented in Table-7. The inhibitory activities of the three RNL extracts against the mycelial growth of *A. niger* were not significantly different from each other, but they had a potential activity compared with the control. In this *in vitro* experiment, miconazole was also used as a standard for antifungal activity and it displayed high activity (15 ± 0.82 mm for *A. fumigatus* and 20 ± 1.87 mm for *A. niger*). The inhibition of mycelial growth of the tested fungi was 81.43–100%, 28.20–78.27%, and 40.17–80.59% for the extract of RWA, R50EOH, and R100EOH, respectively.

**Table-7:** *In vitro* antifungal activity of *Rumex nervosus* leaves extracts on mycelial growth of *Aspergillus fumigatus* (ATCC 28282) and *Aspergillus niger* (ATCC 6275) using food poisonous method

Fungal type/Extracts	R100EOH	R50EOH	RWA	Control (0 mg/mL)	SEM	Probability
Mycelial growth (mm)			
*Aspergillus fumigatus*	23.33±5.0^b^	28.67±9.9^ab^	0.0±0.0^c^	39.00±0.0^a^	4.189	0.004
*Aspergillus niger*	15.33±4.1^b^	17.17±2.7^b^	14.67±1.2^b^	79.00±1.0^a^	4.189	<0.0001
Mycelial growth speed			
*Aspergillus fumigatus*	4.87±0.8	5.72±2.6	0.0±0.0	3.8±0.24	1.37	0.076
*Aspergillus niger*	4.99±0.9^b^	5.28±0.6^b^	4.79±0.5^b^	21.0±0.0^a^	0.416	<0.0001
Growth inhibition (%)			
*Aspergillus fumigatus*	40.17±12.8^b^	28.20±3.6^b^	100.0±0.0^a^	0.0±0.0c	5.025	<0.0001
*Aspergillus niger*	80.59±5.2^a^	78.27±3.4^a^	81.43±1.6^a^	0.0±0.0^b^	2.274	<0.0001

The values are means of three replicates±standard deviation; R100EOH: 100 percent ethanol, R50EOH: 50 percent ethanol, or RWA: water alone are used to extract *Rumex nervosus* leaves. ^a–d^Rows with different letters indicate statistically significant differences (p < 0.05). SEM: Standard error mean

## Discussion

The search for herbal remedies with antimicrobial properties has increased due to their potential benefits in alleviating a variety of chronic and acute infectious diseases. The current investigation focused on a well-known medicinal plant, namely, *R. nervosus*. *Rumex nervosus* is used in Yemen, Saudi Arabia, and other neighboring countries.

It is well known that phenolic compounds are the major antioxidant plant constituents, and it has been found that the antioxidant activity of plant materials is highly correlated with their phenolic compound content. Phenols have also been shown to be effective in the treatment of chronic diseases [44]. Our results show that the leaves of *R. nervosus* have a high content of total phenols. In RNL, the total phenolic content in ethanolic extract was 171.15 mg GAE/g [45]. Based on the above findings, RNL can be considered attractive for food and pharmaceutical applications due to its high content of antioxidants. The presence of physiologically active chemicals, especially phenolic compounds, which vary with genetic diversity and the region from which they were collected, explain the differences in antioxidant activity between samples [46]. The ethanolic extracts from RNL showed antimicrobial activity against selected bacteria and fungi. This could be due to the fact that RNL is rich in bioactive compounds and has higher antioxidant activity.

In this study, the antimicrobial activity of RNL extracts was investigated using three solvents (absolute ethanol, 50% ethanol, and water) against Gram-positive bacteria such as *S. aureus* and *L. monocytogenes*, Gram-negative bacteria (*Salmonella* Typhimurium and *E. coli*), and fungal isolates (*A. fumigatus* and *A. niger*). In addition, the extracts were tested for their antimicrobial activity using ADDM and plate dilution. The different RNL extracts showed different levels of inhibition against the selected bacteria. A positive result was observed when the zone of inhibition had a diameter of >6 mm. Based on the results obtained, the RWA extract had the highest antimicrobial activity against *S. aureus*, which is consistent with the results of this study based on the polarity of the solvents, and it can be assumed that the lower the polarity of the extracts, the higher the antibacterial activity [47]. Although 100% ethanol is a good organic solvent for the extraction of most compounds, it is not as polar as water. The extraction yield, antioxidant properties, radical scavenging activity, and phytochemical content were affected by the polarity of the extraction solvent because the antioxidant compounds in plant parts have a high affinity for more polar solvents compared to nonpolar ones [47]. They found that extraction with more polar solvents (water extract) resulted in a high yield but low flavonoid and phenolic content compared to non-polar solvents. These results indicate that absolute ethanol or a 50% (v/v) water/ethanol mixture are the best solvents for extracting high content of extractable solids from RNL, in contrast to a water solvent alone, which gave a high yield of extractable solids.

The obtained results showed that RWA exhibited significant broad-spectrum antibacterial activity against *S. aureus* and narrow-spectrum antibacterial activity against Gram-negative bacteria using ADDM. The findings of this study are in agreement with a previous Saudi study [47] that found the higher antibacterial activity of polar (aqueous) extract of *R. nervosus* against Gram-positive bacteria (*S. aureus*) and low activity against other Gram-negative bacteria (*E. coli*) using ADDM. In addition, organic extracts such as ethanol and hexane extracts of *R. nervosus* were found to inhibit the growth of microorganisms. However, the organic extract, especially the hexane extract, had lower efficacy against Gram-negative bacteria. Similarly, the methanol extract of *R. nervosus* showed lower efficacy against Gram-negative bacteria [48]. Al-Nowihi *et al*. [21] showed that the ethanolic and methanolic extracts of *R. nervosus* have high antimicrobial activity against the bacteria *S. aureus* and *E. coli* and against the fungus *Candida albicans* using ADDM, which means that it has the potential to be used as an antimicrobial agent. Although the results of this study support the findings of Al-Nowihi *et al*. [21] on the high activity of *R. nervosus* against *S. aureus*, our results contradict their findings with respect to *E. coli*, where they demonstrated that *R. nervosus* had a strong growth inhibitory effect on *E. coli* among Gram-negative bacteria in both ethanolic and methanolic solvents. However, the obtained result regarding the effect of *R. nervosus* extracts on *E. coli* is in agreement with other studies, which showed that the extract of *R. nervosus* had the lowest anti-*E. coli* activity [49, 50].

In this study, RWA showed superior antimicrobial activity against *S. aureus*, while R50EOH showed superior activity against *E. coli* compared to other *Rumex* extracts. However, *R. nervosus* extracts showed low activity against other organisms, such as *Salmonella* Typhimurium and *L. monocytogenes*. Since ethanol is a colorless volatile liquid, the antibacterial activity of *R. nervosus* extract seems to be more inhibited in ethanol than in methanol and appears to be more inhibitory for Gram-positive bacteria than for Gram-negative bacteria [21].

In this case, the antibacterial activity of *R. nervosus* extracts was limited to Gram-negative bacteria, although it showed a zone of inhibition against all Gram-negative bacteria, Gram-positive bacteria, and fungal strains. With the exception of *S. aureus*, *R. nervosus* was found to be less effective against Gram-negative and Gram-positive bacteria, and not effective against fungi when used with ADDM. The MIC was used to determine the effectiveness of the extracts on the bacterial and fungal strains tested. Only organisms that had a zone of inhibition in the previous ADDM and were sensitive to the herbal extracts were tested for MIC [22]. All the extracts tested were found to have antimicrobial activity. The MIC of the R100EOH extract against *Salmonella* Typhimurium and *E. coli* was 0.20 mg/mL, but against *S. aureus* was 0.32 mg/mL.

Here, the extracts of *R. nervosus* inhibited Gram-positive and Gram-negative bacteria, with no effect on *A. fumigatus* and *A. niger*, supporting reports from Ethiopia and Saudi Arabia against *S. aureus*, *Pseudomonas aeruginosa*, and *Streptococcus pyogenes*, but not *C. albicans* [22, 51].

*Rumex nervosus* extracts showed the highest DIZ (20 mm) against the genus *Salmonella* compared to *Plantago lanceolata*, *Lepidium sativum*, and *Solanum incanum* [52]. They found that the MIC and MBC of *R. nervosus* extracts against *S. aureus* were 1.56 mg/mL. *Rumex nervosus* also showed the highest MIC against many organisms tested [22].

Several species of *Rumex* (family *Polygonaceae*) have antimicrobial activity. However, ADDM results exhibited that the EOs have a broad spectrum of antimicrobial activity against pathogenic bacteria. The spectrum of antimicrobial activity varies due to the presence of different minor and major constituents in EO of each plant, which is due to the extraction method, plant species, plant part, microbial strains, geographical origin and environmental conditions, test conditions, test procedures and concentrations used, etc. [53–55]. In studying the antimicrobial activities of 22 EOs, Yousef and Tawil [56] discovered discrepancies between ADDM and dilution tests. Rios *et al*. [57] found that ADDM is not suitable for samples that are difficult to dissolve in water or for nonpolar samples. In addition, there was no correlation between diffusivity and antimicrobial activity nor between MIC values and growth inhibition diameter. The ADDM, on the other hand, was ideal for screening because it has a small sample size and can test multiple compounds against a single microorganism. A dilution method was recommended to determine MIC and MBC with the greatest precision and suitability for nonpolar samples such as EO [57, 58]. Therefore, the microplate dilution assay was used in this study to evaluate the efficacy of antimicrobial activity. Moreover, the R100EOH minimally inhibited the selected bacteria at a much lower concentration than the 50% ethanol extract and the water extract, except for *L. monocytogenes*. In addition, the R100EOH had a lethal effect (MBC) on most of the bacteria studied at a much lower concentration than the R50EOH extract and the RWA. However, the RWA showed the highest lethal effect on *L. monocytogenes*. This extract proved to be both inhibitory and bactericidal. As indicated by the lowest MIB and MBC values, *Salmonella* Typhimurium and *E. coli* appeared to be the most sensitive bacteria to RNL. As a result, *Salmonella* Typhimurium and *E. coli* were more sensitive to R100EOH than *S. aureus* and *L. monocytogenes*.

These data indicate that Gram-negative bacteria were more sensitive to RNL extracts than Gram-positive bacteria. Nevertheless, comparisons of bacterial susceptibility may differ. This is due not only to the antimicrobial agents in EO but also to the diversity of microorganisms tested [53, 59]. Another factor that may have contributed to the different results was the type of media used to test antimicrobial activity. The growth conditions for the bacteria may differ when different media are used. For example, Khakhanang *et al*. [58] used a nutrient broth medium in their study, Kasimala *et al*. [60] used a Mueller-Hinton agar medium in their study, and a nutrient agar medium was used in this study. These aforementioned factors may be responsible for the conflicting results in each case. However, the promising antimicrobial properties of EO, especially the extracts of RNL reported here, could recommend their use as a substitute for antibiotics in the treatment of infectious diseases in animals. However, the toxicity and safety of the use of these extracts must be considered. In addition, the results obtained indicate that RNL has antimicrobial activity against bacteria and fungi, which means that they could be used as antimicrobial agents.

The minimum inhibitory concentration of RNL ranged from 0.20 to 10.41 mg/mL for the absolute ethanol extract, 0.82–15.63 mg/mL for the 50% ethanol extract, and 6.51–7.81 mg/mL for the water extract. The MBC of RNL ranged from 0.40 to 20.81 mg/mL for the absolute ethanol extract, 1.63–31.26 mg/mL for the 50% ethanol extract, and 13.02–15.62 mg/mL for the water extract. This is somehow similar to the report of Liang *et al*. [61], who observed MBC values ranging from 20 to 160 mg/mL for ethanol extracts. Moreover, MIC and MBC of RNL extracts ranged from 0.20 to 13.02 for Gram-negative bacteria and from 0.32 to 31.26 mg/mL for Gram-positive bacteria. In contrast, Alhajj *et al*. [33] reported that MIC and MBC values ranged from 7.8 to 31.2 mg/mL for both types of bacteria.

Traditional healers could prepare their medicines with water because they do not have access to more lipophilic solvents [62]. This is of concern because it is possible that the healers do not extract all the active ingredients contained in the plant. Dosage is a critical factor in choosing which solvent to use. Several studies have shown that organic solvents are superior to aqueous extracts in terms of antibacterial activity [61, 63, 64]. Thus, the dosage would be higher if a water extract is used, while the same dosage could be dangerous if a lipophilic solvent extract is used. For rural communities and informal settlements, it is beneficial to determine the antibacterial and antifungal properties of plants used in traditional medicine.

Several researchers have used this method to evaluate the antifungal activity of herbs [65, 66]. According to Gwa and Ekefan [67], some plant extracts (neem, tobacco, ginger, papaya, black pepper, and Mancozeb) have fungicidal activity against yam disease caused by *A. niger*. The results of food poisoning with RNL extracts suggest that RNL extracts can be considered as effective natural agents to inhibit mycelial growth compared to control.

The chemical composition of the methanolic RNL extracts in this study was investigated by HPLC and GC–MS, which led to the identification of gallic acid and 13 compounds, respectively. In the HPLC results, gallic acid has the highest peak area fraction. The presence of various phenolic acids and flavonoids in mushroom ethyl acetate extracts was quantified by HPLC [68]. Due to the negative effects of free radicals and toxic byproducts of their metabolism on various metabolic processes [69] during the immune response of host cells to microbial invasion, phenolic compounds may play an important role in the defense against microbial infections and in averting tissue damage and cytotoxicity [70].

Our GC–MS results for phytochemical analysis show that saturated octadecanoic acid and unsaturated octadecanoic acid, also known as 9, 2-octadecadienoic acid or omega-6 unsaturated fatty acid, have the highest peak area fraction. In addition, other fatty acids, including pentadecanoic acid (pentadecylic acid), hexadecanoic acid (also known as palmitic acid), and other phytochemicals, including methoxyphenyl-oxime, cyclotrisiloxane, 5-methyl-2-phenylindolizine, and 1,2-bis(trimethylsilyl) benzene, were present in moderate amounts. The tested bacteria and fungi can be killed or inhibited by the powdered leaves of *R. nervosus*. At low concentrations, the powder extracts of the leaves have been found to have antifungal and antibacterial properties. The presence of a secondary plant compound in RNL powder is responsible for its activity. According to Krishnaveni *et al*. [71], GC–MS analysis of phytochemicals and fatty acid profiles of natural plants have antimicrobial activity in killing bacteria and fungi. Basil hexane extract and ethanol extract had the best antibacterial activity and lowest MIC concentration. Therefore, they were selected as the most effective antibacterial extracts for GC/MS analysis to determine their chemical constituents [72]. In the GC–MS analysis of the methanolic extract of RNL, 13 compounds were detected, whose activity and mode of action against pathogenic microbes, either alone or in combination with other antimicrobial substances, need to be further investigated.

## Conclusion

The three extracts of RNL herb had varying degrees of inhibitory activity against the selected bacteria and were resisted by the tested fungi. R100EOH showed good bacteriostatic and bactericidal activity. This antimicrobial activity can be attributed to the presence of the main bioactive constituents of RNL, especially gallic acid. It is concluded that RNLs can be developed as natural alternatives for further use in food preservation to prevent microbial contamination in the food industry and to extend the shelf life of food as novel preservatives that can replace synthetic bactericidal or fungicidal agents. Field studies are recommended to test the bioactive extract against the selected bacteria or fungi. In addition, the results of this study would help to develop new antimicrobial agents in the future.

## Authors’ Contributions

MAA and MMQ: Conceptualized and designed the study and performed the *in vitro* experiments. MMQ and AHA: Analyzed the data, wrote, and edited the manuscript. SIA: Supervised the study. AP, MAA, and SIA: Corrected the manuscript. All authors have read and approved the final manuscript.
